# Computerized Evaluation of Attention, Learning-Memory, and Executive Function in People with Disability Caused by Injury: A Pilot Study

**DOI:** 10.3390/jcm14072153

**Published:** 2025-03-21

**Authors:** Sandra Rute-Pérez, Carlos Rodríguez-Domínguez, Noelia Sáez-Sanz, Miguel Pérez-García, Alfonso Caracuel

**Affiliations:** 1 Mind, Brain and Behavior Research Center (CIMCYC), University of Granada, 18011 Granada, Spain; 2 Department of Developmental and Educational Psychology, Faculty of Education Sciences, University of Granada, 18011 Granada, Spain; 3 Department of Computer Languages and Systems, Faculty of Education, Economy and Technology of Ceuta, University of Granada, 18011 Granada, Spain; 4 Department of Developmental and Educational Psychology, Faculty of Education Sciences, University of Valladolid, 47002 Valladolid, Spain; 5 Department of Personality, Assessment and Psychological Treatment, Faculty of Psychology, University of Granada, 18011 Granada, Spain

**Keywords:** acquired brain injury, cognitive assessment, computerized assessment, VIRTRAEL

## Abstract

**Background/Objectives**: This study aims to validate the tests in the VIRTRAEL assessment module for individuals in the chronic stage of Acquired Brain Injury (ABI). **Methods**: A total of 32 participants (81.3% male) were assessed, including 20 individuals who had experienced a stroke (62.5%) and 12 who had experienced a traumatic brain injury (TBI). Cognitive functions such as attention (d2 Attention Test and Digit Subscale of the WAIS-III), learning-memory (HVLT-R and Letters and Numbers Subscale of the WAIS-III), and executive function (Similarities and Matrices Subscales of the WAIS-III and the Zoo Map Test, version 1 of the BADS Battery) were evaluated using standardized paper-and-pencil tests and computerized tests from the VIRTRAEL online platform. Convergent and divergent correlation analyses were conducted using SPSS version 28. **Results**: Significant correlations were observed between scores in both formats (ranging from r = 0.408 in planning to r = 0.818 in delayed verbal recall), except in reasoning and verbal memory recognition. **Conclusions**: The tests from the free online platform VIRTRAEL demonstrate adequate validity for the cognitive assessment of individuals with ABI. This suggests that VIRTRAEL is a valuable, accessible tool for monitoring cognitive status, potentially benefiting health, education, and social and occupational reintegration.

## 1. Introduction

Acquired Brain Injury (ABI) is a significant health concern worldwide. It refers to a sudden injury to brain structures occurring after birth, leading to various impairments [1]. The most common causes of ABI are cerebrovascular accidents (CVA), particularly ischemic strokes, and traumatic brain injuries (TBI), both of which are leading causes of adult disability [2,3].

Recent research indicates that, between 1990 and 2021, the global incidence of stroke increased by 70%, with approximately 11.9 million new cases in 2021. Prevalence and incidence rates were 819.5 and 92.4 cases per 100,000 people, respectively. The disability-adjusted life years (DALYs) rate was 837.4 per 100,000 people, indicating that millions live with stroke-related disabilities. Moreover, a 10% increase is expected by 2040 [4]. Additionally, TBI affects an estimated 64–74 million people globally each year [5]. In 2019, 27.16 million new cases were reported, with age-adjusted incidence and prevalence rates of 346 and 599 per 100,000 population, respectively. Moreover, TBI was the cause of 7.08 million years lived with disability, with an age-adjusted rate of 86.5 per 100,000 people [6].

The significance of these figures is reflected in the impact of ABI, which not only affects individuals but also has repercussions for their families and social environments. Cognitive, emotional, and behavioral alterations [7,8,9,10] often result in reduced independence, autonomy, and life expectancy. Among the most prevalent cognitive deficits are problems with attention, learning, memory, and decision-making [11,12,13,14], which are associated with an increased risk of neurodegenerative conditions such as Alzheimer’s disease in this population [15,16,17,18]. For example, a systematic review and meta-analysis found a significant association between a history of stroke and an increased risk of developing Alzheimer’s disease [18]. TBI-induced neurovascular injury accelerates β-amyloid (Aβ) production and perivascular accumulation, which can initiate cerebrovascular pathology. This plays a causal role in the development of multiple forms of neurodegeneration, including AD-like dementias [19].

Numerous studies have sought to find measures capable of reversing the damage and improving the quality of life for both affected individuals and their families [20,21,22]. Some cognitive intervention programs have reported significant benefits and positive results in reducing cognitive impairment [23,24]. For instance, a recent systematic review underscores the effectiveness of cognitive training in improving verbal memory, executive function, and performance in instrumental activities of daily living (IADL) in people with ABI, even when applied after 12 months post-injury [25].

However, prevalence rates indicate that a high percentage of individuals experience chronic cognitive impairments and functionality problems [7,15,26,27,28]. Many improvements achieved in subacute rehabilitation centers often decline in the months post-discharge due to limited resources and motivation for continued self-directed rehabilitation and adaptation. Additionally, healthcare system deficiencies, coupled with the severity of ABI, mean that many people continue to require care, imposing substantial costs on families and healthcare services [29,30,31,32]. Studies tracking ABI-related disabilities over periods of up to 20 years indicate a lack of significant long-term improvement [33], likely because many individuals are left helpless and excluded from healthcare services [34]. This is one of the reasons why ABI has been described as the silent epidemic, with many affected individuals becoming invisible to healthcare systems.

The rise of digital technologies has facilitated innovative alternatives for supporting and caring for individuals with ABI [35,36]. For instance, cognitive training tools activate brain plasticity and can enhance cognitive performance, extending to improved functioning in activities of daily living [23,37,38]. Alongside computerized intervention programs, reliable assessment tools are essential for providing valid measures of cognitive status, determining where improvements are needed, adjusting interventions, and evaluating their effectiveness [39,40]. Computerized neuropsychological assessment tests offer potential advantages over standardized pencil-and-paper tests, including (1) the ability to assess a large number of people quickly and easily, (2) broader accessibility, (3) precise, time-efficient measurement, and (4) reduced administration costs [41,42,43,44]. However, concerns regarding the psychometric properties of certain computerized batteries, such as test–retest reliability and sensitivity, remain an issue [45].

VIRTRAEL is a free online platform designed for evaluating cognitive processes, targeting attention, learning-memory, and executive functions (working memory, reasoning, and planning). Originally developed for older adults, its initial version has shown validity for assessment in this population. It has also demonstrated effectiveness in improving key cognitive functions, including attention, working memory, and planning abilities [46,47]. Based on the hypothesis that this platform will also be valid and useful for assessing the ABI population, this study aimed to preliminarily validate the tests contained in the VIRTRAEL assessment module for individuals with chronic-phase ABI.

## 2. Materials and Methods

### 2.1. Design

The study employed a quasi-experimental, correlational design incorporating concurrent and divergent validation.

### 2.2. Participants

Participants were recruited from two ABI patient associations in Granada, Spain. The total sample consisted of 32 adults (81.3% male) with an average age of 56.22 years (SD = 14.08), of whom 20 (62.5%) had suffered a stroke and 12 had a TBI. All participants attended the center occasionally for organized group activities (mainly socioemotional) but did not receive neuropsychological or motor rehabilitation sessions at the time of the study.

The study’s inclusion criteria consisted of: (a) diagnosed with ABI due to stroke or TBI; (b) a minimum of 6 months post-injury; (c) preserved or recovered reading and writing abilities; (d) sufficient motor function in the dominant hand for computer use; (e) score of ≥21 on the Spanish adaptation of the Mini-Mental State Examination (MMSE) to ensure cognitive ability that ruled out the presence of dementia [48].

### 2.3. Instruments

All participants were individually assessed using the Spanish version of various standardized neuropsychological tests and the VIRTRAEL assessment module to conduct the validation. The functions assessed were attention, learning and verbal memory, working memory, reasoning, and planning.

#### 2.3.1. Standardized Neuropsychological Tests

- d2 Test of Attention [49], which assesses selective attention and concentration. The final scores comprise the total number of correct responses and errors, from which a concentration index (hits minus commission errors) is calculated.
- Digit Span subtest from the Wechsler Adult Intelligence Scale-III (WAIS-III) [50]. This test evaluates auditory attention and working memory. The final score is the total number of correctly repeated series.
- Hopkins Verbal Learning Test-Revised (HVLT-R) [51], Form A, which assesses verbal learning and memory. The study included the following indices: Trial 3 (number of words recalled in the third learning trial); Learning T (total words learned across three trials); Delayed recall (number of correctly recalled words after a delay); and Recognition (number of accurately identified words).
- Letters–Numbers Sequencing (L&N) from the WAIS-III [50]. This test assesses working memory. The score is determined by the number of accurately repeated sequences.
- Similarities subtest from the WAIS-III [50]. This evaluates semantic reasoning. The total score is calculated by summing all correctly completed items.
- The Matrix Reasoning Subtest from the WAIS-III [50] assesses abstract reasoning. The score is determined by the overall number of accurate responses.
- Zoo Map Test (Version 1) from the Behavioral Assessment of the Dysexecutive Syndrome (BADS) battery [52]. This test evaluates planning abilities. The final score is obtained by deducting the total errors from the number of accurately completed sequences. Errors include revisiting locations more than once, deviating from the designated route, failing to mark the route with a solid line, and visiting inappropriate locations.

#### 2.3.2. Computerized Neuropsychological Tests

VIRTRAEL (“Virtual Training for the Elderly”, n.d.) (http://www.everyware.es/webs/virtrael/#home, accessed on 5 September 2024) is a freely accessible online platform developed for cognitive evaluation and stimulation. Originally developed as a software application named PESCO, it was validated for elderly individuals without dementia through a project funded by a regional public agency [46]. Following its validation, it was converted into a web-based format to facilitate access through any Internet-connected device [47,53].

VIRTRAEL comprises 13 sessions: two pre-assessment sessions, nine stimulation sessions, and two post-evaluation sessions. For this study, participants completed only the first two sessions of the evaluation module, each lasting between 40 and 60 min.

- Session 1: This session includes a marksmanship test, sociodemographic data collection, the Barthel Index for Basic Activities of Daily Living [54], the Lawton–Brody Instrumental Activities of Daily Living Scale [55] and pre-stimulation cognitive assessment tests:
  ◦ Semantic series: A verbal reasoning task that establishes relationships between word categories. On each screen, a set of words is displayed, and the user must identify the one that does not belong to the group.
  ◦ Logical series: A reasoning exercise involving figures designed to assess perceptual skills. Different screens display figure sequences that follow a specific pattern. To complete the sequence, the user must determine the underlying criterion and select the appropriate figure from the options provided at the bottom of the screen.
- Session 2: Pre-stimulation Cognitive Screening:
  ◦ List of Words (short- and long-term memory): This is designed to assess learning and verbal memory. Participants are presented with a list of 12 words in blue for 60 s and are then asked to recall and write down as many as possible. This process is repeated two more times with the same list. Afterward, a different list of words, presented in red, is shown for 60 s, and participants are asked to recall and record them. This red list is shown only once. After approximately 20 min, participants are asked to recall words from the blue list. First, they write down as many as they remember. Then, a recognition task is presented, in which words from both lists, as well as additional unrelated words, appear on separate screens. The participant must indicate whether each word appeared in the blue list by selecting “yes” or “no”. Each word, along with the “yes/no” buttons, appears on a different screen (extending the list to several pages).
  ◦ Numbers: This exercise is designed to measure attentional capacity. Participants see a sequence of numbers on a blackboard, which they must replicate in the same order. The exercise starts with short sequences, which increase in length as the participant progresses. Two sequences are performed per level; if at least one is correct, the participant advances to the next level. The maximum number of levels is nine, and the exercise ends when the participant makes two errors at the same level.
  ◦ Numbers–Vowels: This exercise aims to measure working memory using an interface similar to the previous exercise. Participants memorize vowels and numbers that appear sequentially in random order and must input them in a structured manner: first, the numbers in ascending order, followed by the vowels in alphabetical order. For example, if “a, 5” appears, the participant must enter “5, a”. If “8, e, 2, u” appears, they must enter “2, 8, e, u”.
  ◦ Pyramids: This exercise measures the user’s sustained attention span over a 5 min period. Participants are shown panels containing various images of pyramids—small, large, rotated, facing forward, with or without doors. Their objective is to identify images that contain one large pyramid and two small pyramids turned with a door on the sunny side before the allotted time runs out.
  ◦ Parcel delivery: This exercise is designed to evaluate planning and organizational skills (to achieve planned goals). Participants act as package delivery drivers, picking up and delivering color-coded packages while following an optimal route and avoiding unnecessary detours. Before starting, they receive instructions on how to navigate the city. The interface continuously displays the remaining fuel level and the packages being carried. The task list remains visible throughout, showing the three packages that need to be picked up and the three that need to be delivered, eliminating the need for memorization.

VIRTRAEL automatically records the time spent on each activity, as well as accuracy and failure rates. At the beginning of each task, an avatar assistant explains the objectives and steps of the exercises. Additionally, all exercises include a preliminary trial to ensure that the participant understands the instructions.

A demo version of VIRTRAEL is available: https://virtrael-demo.web.app/exercises (accessed on 5 September 2024).

### 2.4. Procedure

Participants were briefed about the study, and all volunteers gave written informed consent, following approval from the Ethics Committee of the University of Granada (certificate no. 364/2010). Subsequently, each participant was assigned individual assessment sessions. The validation procedure was carried out in two phases:

- Phase 1. This consisted of a single session of approximately one hour, during which participants were assessed on attention, verbal memory, working memory, reasoning, and planning using standardized tests.
- Phase 2. This phase consisted of two sessions, each approximately 45 min long. Participants completed seven cognitive tasks from the VIRTRAEL assessment module in these sessions, corresponding to the cognitive domains previously evaluated with paper-and-pencil tests. Each participant was assigned a unique username and password to access the platform.

All data were securely archived at the Mind, Brain, and Behavior Research Center (CIMCYC) at the University of Granada.

### 2.5. Statistical Analysis

To determine the association between the scores from the computerized tests and the corresponding standardized tests (see Table 1), the Pearson correlation coefficient was used when the data distribution met normality criteria according to the Shapiro–Wilk test (attention, learning and verbal memory, working memory, and planning). In cases where normality was not met (reasoning), the Spearman correlation coefficient was applied. Correlations were also conducted between the VIRTRAEL assessment tests and the daily living activity scales. All statistical analyses were conducted using SPSS version 28.

## 3. Results

Table 2 displays participants’ results for both the standardized assessments and the corresponding computerized tests from VIRTRAEL. The results reveal significant correlations between the two test formats across all measured domains, except for reasoning tests and the recognition index of verbal memory tests. The weakest significant correlation was observed in planning test scores (r = 0.408), while the strongest was found in delayed verbal recall indices (r = 0.818).

To determine the relationship between cognitive performance and daily living activities, correlations were conducted between the VIRTRAEL assessment tasks and the daily living activity questionnaires. The results revealed moderate and significant correlations between the total score of the Lawton–Brody Instrumental Activities of Daily Living Scale and the tests of attention (Digits: r = 0.693, *p* = 0.009; Pyramids: r = 0.489, *p* = 0.008), working memory (Numbers and Vowels: r = 0.528, *p* = 0.007), and planning (Parcel delivery: r = 0.502, *p* = 0.009). No significant correlations were found for the remaining variables, nor with the Barthel Index for Basic Activities of Daily Living.

To further assess divergent validity and ensure comprehensive analysis in the concurrent validation, correlation analyses were conducted between all VIRTRAEL test scores and all standardized test scores used (Table 3 and Table 4). Each VIRTRAEL index or test showed a stronger association with its corresponding standardized test variable, except for Numbers–Vowels Sequence, Semantic Series, and Parcel Delivery (Table 4). However, when comparing standardized tests among themselves, similar patterns emerged. The Letters–Numbers subscale showed stronger correlations with d2 (r = 0.673), while the Matrix Reasoning subscale correlated more strongly with d2 (r = 0.577) and the Letters–Numbers Sequencing subscale (r = 0.567). Additionally, the Zoo Map test showed moderate to strong correlations across multiple standardized tests: d2CON (r = 0.366), HVLT-R-trial3 (r = 0.401), HVLT-R-LearT (r = 0.706), HVLT-R-delayed (r = 0.507), HVLT-R-recog (r = 0.663), Letters–Numbers Sequencing (r = 0.437), Matrix Reasoning (r = 0.638), and Similarities (r = 0.427) (Table 4).

## 4. Discussion

This study aimed to establish the preliminary concurrent validity of the assessment module tests within the free VIRTRAEL platform for individuals with chronic-phase Acquired Brain Injury (ABI). The comparison results suggest moderate to high validity [56] in evaluating attention, verbal learning and memory, working memory, and abstract reasoning and planning in this population.

Previous research has suggested that, in psychological assessment, the average effect size of correlations between computerized and standardized tests is around 0.3 [57]. In this study, performance on VIRTRAEL computerized tasks assessing learning and verbal memory showed strong correlations above 0.8 with their standardized counterparts (total learning: r = 0.806, *p* < 0.001; delayed recall: r = 0.818, *p* < 0.001). Similarly, strong effects were observed for working memory and concentration tests. Moderate correlations above 0.4 were found in abstract reasoning and planning. However, the study hypothesis was not fulfilled for the semantic reasoning test and the long-term word list recognition index, as no significant correlations were found with the corresponding standardized tests.

In addition to strong concurrent validity (high associations between indices measuring similar constructs), full validity also requires divergence—meaning certain indices should not be highly correlated if they assess distinct constructs. The results showed expected multiple associations across different test formats, as cognitive test performance often involves coordinating multiple functions [58,59]. Our findings indicate that most VIRTRAEL tests that demonstrated concurrent validity also showed divergent validity, presenting correlation patterns with standardized tests similar to expected norms. Notably, the Parcel Delivery test demonstrated better divergent validity than the Zoo Maps test, its corresponding standardized counterpart. This stronger concurrent–divergent validity of the Parcel Delivery test is based on the fact that scores on this test showed no correlation with the HVLT-R memory indices or the Similarities test, whereas Zoo Map did, despite no a priori expectation of such correlations.

In three VIRTRAEL tests (Number–Vowel Sequence, Logic Series, and Parcel Delivery), a stronger correlation was found with an index initially considered non-equivalent. However, these unexpected correlations were only marginally higher than those with the expected indices, and similar phenomena occurred in the standardized tests, often with greater magnitude.

Previous studies have highlighted the advantages of computerized cognitive assessment over traditional neuropsychological tests, including greater accessibility, faster and more precise measurements, structured administration, immediate feedback, and reduced errors from therapist involvement [60,61]. The results of this study regarding the validity of VIRTRAEL are consistent with those from other computerized platforms [62]. However, the validity (and thus clinical utility) of most available computerized tests has not been studied or remains questionable [43,63], with many entailing substantial costs for families. The successful validation of most VIRTRAEL tests in this study has clinical significance, supporting the use of a freely available and accessible tool for monitoring the cognitive status of individuals with ABI, aiding their health, education, and social and occupational reintegration.

Based on the correlation results between the total score of the IADL questionnaire and cognitive function in attention, working memory, and planning, it is possible to interpret that functionality in complex daily activities is strongly influenced by executive and working memory domains. Previous evidence suggests that executive functions, particularly those involved in sustained attention, cognitive flexibility, and planning, are key determinants in preserving functional autonomy [64]. Previous studies have documented that impairments in these functions are associated with greater difficulty in performing instrumental activities of daily living, which may indicate progression toward more severe stages of cognitive decline [65]. Furthermore, the strong association between attention and instrumental activities of daily living can be explained by the fundamental role of this function in regulating and coordinating daily tasks that require the simultaneous processing of multiple sources of information [64]. Similarly, working memory enables the temporary retention and manipulation of information, which is essential for the proper execution of activities requiring sequential steps and informed decision-making [65]. Taken together, these findings support the hypothesis that the integrity of executive functions and working memory is crucial for maintaining functional independence and highlight the importance of early neuropsychological assessment in populations at risk of cognitive decline. The lack of correlations between cognitive functioning and basic daily living skills could be due to the fact that the latter are more closely associated with motor function [66,67].

A limitation of this study is the small sample size, which restricts the generalizability of the findings. This limitation is compounded by difficulties in accessing participants through associations and residences. Due to limited resources and a lack of coordination among services, individuals with chronic-phase ABI often experience isolation, making it challenging to locate them or coordinate evaluations with their families. Another limitation of this study is the uneven gender distribution of our sample, with 81.3% of participants being men. This imbalance is due to the greater availability of male participants at the reference center where the study was conducted and aligns with the epidemiological distribution of the condition, as men have a higher prevalence of ABI compared to women. However, future studies will aim for a more balanced sample to explore potential sex-related differences. Another limitation is the lack of subgroup analysis between participants with TBI and those with stroke, which may have masked specific differences based on etiology. However, the primary goal of this pilot study was to evaluate the feasibility of the protocol and the applicability of cognitive assessment tools in individuals with ABI. Given its exploratory nature, subgroup comparisons were not conducted, as the focus was on assessing the study design and data collection process before a larger-scale investigation. Moreover, subgroup analysis requires a larger sample size to ensure statistically robust findings. Future studies with more participants will allow for a more detailed examination of differences between TBI and stroke and their impact on cognitive outcomes. Finally, although the participants in our study did not receive neuropsychological or motor rehabilitation sessions at the time of evaluation, it is important to consider that many individuals with stroke or traumatic brain injury have undergone some form of prior rehabilitation throughout their recovery process. This rehabilitation may have included motor or cognitive training alone or combined. In this regard, recent research has highlighted the interdependence between cognitive and motor functions in dual-task paradigms, suggesting that deficits in one of these areas may influence performance in the other [68]. Although our study did not include a specific dual-task assessment, the possible influence of previous rehabilitation experiences on our participants should be considered as a factor that may have affected their performance. Future research could further explore this interaction by evaluating the impact of dual-task-based interventions in this population.

## 5. Conclusions

In conclusion, the Pyramids, List of Words, Numbers, Number–Vowel Sequence, Logical Series, and Parcel Delivery tests of VIRTRAEL demonstrate adequate validity for cognitive assessment in individuals with acquired brain damage. This makes VIRTRAEL a valid, low-cost, and accessible tool for individuals with limited financial resources or difficulty traveling to specialized centers. However, the Semantic Series test and the recognition index of the List of Words task did not demonstrate satisfactory validity.

## Figures and Tables

**Table 1:** Correspondence of the comparison between standardized tests and VIRTRAEL.

Cognitive Domain	Standardized Tests	VIRTRAEL Tests
Attention	d2 test	Pyramids
	Digits (WAIS-III)	Numbers
Learning and verbal memory	HVLT-R	List of Words
Working memory	Letters and Numbers (WAIS-III)	Numbers and Vowels
Reasoning	Matrix (WAIS-III)	Logical Series
	Similarities (WAIS-III)	Semantic Series
Planning	Zoo maps-version 1 (BADS)	Parcel delivery

Note: WAIS-III = Wechsler Adult Intelligence Scale-III; HVLT-R = Hopkins Verbal Learning Test–Revised; BADS = Behavioral Assessment of the Dysexecutive Syndrome; d2 = d2 Attention Test d2.

**Table 2:** Means and correlation coefficients between the corresponding indexes of the standardized tests and those of the VIRTRAEL computerized tests.

Cognitive Domains	Standard Format		Computerized Format VIRTRAEL		r/Rho (*p*)
	Index (Test)	Mean (SD)	Index (Test)	Mean (SD)	
Attention	Hits (d2)	110.22 (42.46)	Hits (Pyramids)	82.75 (35.37)	0.783 (*p* < 0.001)
	Concentration (d2)	107.72 (44.49)	Concentration (Pyramids)	78.94 (38.66)	0.780 (*p* < 0.001)
	Span attention (Digits, WAIS-III)	7.63 (2.18)	Span attention (Numbers)	8.03 (3.10)	0.711 (*p* < 0.001)
Learning-verbal memory	Trial 3 learning (HVLT-R)	8.66 (2.50)	Trial 3 learning (List of Words)	8.13 (2.68)	0.771 (*p* < 0.001)
	Total learning (HVLT-R)	22.78 (6.78)	Total learning (List of Words)	20.47 (8.35)	0.806 (*p* < 0.001)
	Delayed recall (HVLT-R)	7.41 (2.90)	Delayed recall (List of Words)	5.70 (3.14)	0.818 (*p* < 0.001)
	Recognition (HVLT-R)	10.69 (1.71)	Recognition (List of Words)	10.71 (1.79)	0.113 (*p* = 0.546)
Working memory	Letters and Numbers (WAIS-III)	7.13 (2.83)	Numbers and Vowels	7.87 (3.81)	0.662 (*p* < 0.001)
Reasoning	Matrix (WAIS-III)	10.81 (5.57)	Logical Series	2.53 (1.31)	0.444 (*p* = 0.011)
	Similarities (WAIS-II)	16.75 (5.68)	Semantic Series	4.25 (0.84)	0.207 (*p* = 0.255)
Planning	Zoo maps-version 1 (BADS)	−0.94 (5.64)	Parcel delivery	5.64 (1.11)	0.408 (*p* = 0.02)

Note: WAIS-III = Wechsler Adult Intelligence Scale-III; HVLT-R = Hopkins Verbal Learning Test–Revised; BADS = Behavioral Assessment of the Dysexecutive Syndrome; d2 = d2 Attention Test.

**Table 3:** Results of the correlation between all tests and indices of attention, learning, and verbal memory in the computerized and standardized formats (coefficients between tests considered equivalent are in bold).

	Attention				Memory							
	Attentional Span		Concentration		Learning				Long-Term Memory			
	Standard	VIRTRAEL	Standard	VIRTRAEL	Standard	VIRTRAEL	Standard	VIRTRAEL	Standard	VIRTRAEL	Standard	VIRTRAEL
	Digits	Numbers	d2-CON	PYR-CON	HVLT-R-trial3	LW-trial3	HVLT-R-LearT	LW-LearT	HVLT-R-Delayed	LW-Delayed	HVLT-R-Recog	LW-Recog
d2-CON	0.413 *	0.544 **	-	0.780 **	0.403 *	0.560 **	0.407 *	0.3 ^a^	0.410 *	0.404 *	0.378 *	−0.226
HVLT-R-trial3	0.155	0.164	0.403 *	0.452 **	-	0.771 **	0.960 **	0.811 **	0.855 **	0.844 **	0.668 **	0.118
HVLT-R-LearT	0.171	0.207	0.407 *	0.472 **	0.960 **	0.706 **	-	0.806 **	0.833 **	0.828 **	0.677 **	0.038
HVLT-R-delayed	0.17	0.136	0.410 *	0.379 *	0.855 **	0.686 **	0.833 **	0.689 **	-	0.818 **	0.786 **	0.249
HVLT-R-recog	0.07	0.216	0.378 *	0.374 *	0.668 **	0.543 **	0.677 **	0.468 **	0.786 **	0.664 **	-	0.113
Digits	-	0.711 **	0.413 *	0.354 *	0.155	0.238	0.171	0.118	0.017	0.124	0.216	0.07
L&N	0.677 **	0.645 **	0.673 **	0.690 **	0.376 *	0.397 *	0.363 *	0.223	0.443 *	0.344 ^b^	0.449 *	−0.257
Matrix	0.414 *	0.591 **	0.577 **	0.465 **	0.347	0.444 *	0.294	0.202	0.396 *	0.418 *	0.366 *	0.097
Similarities	0.429 *	0.317	0.428 *	0.451 **	0.366 *	0.562 **	0.468 **	0.498 **	0.296	0.321	0.224	−0.214
Zoo maps	0.238	0.333	0.366 *	0.404 *	0.401 *	0.474 **	0.706 **	0.311 ^b^	0.507 **	0.515 **	0.663 **	0.17

Note: * *p* ≤ 0.05; ** *p* ≤ 0.001; ^a^ tendency to significance *p* = 0.09; ^b^ tendency to significance *p* = 0.08; d2-CON = Test d2 concentration index; HVLT-R = Hopkins Verbal Learning Test–Revised (trial3 = trial 3; LearT = learning; recog = recognition; L&N = Letters and Numbers; LW = VIRTRAEL Word List (trial3 = trial 3; LearT = learning; recog = recognition).

**Table 4:** Correlation results between all tests and indices of executive function in the computerized and standardized formats (in bold are the coefficients between concurrent tests, underlined when the highest correlation does not occur with the test considered a priori as equivalent).

	Executive Function							
	Working Memory		Reasoning				Planning	
	Standard	VIRTRAEL	Standard	VIRTRAEL	Standard	VIRTRAEL	Standard	VIRTRAEL
	L&N	N&V	Matrix	S. Log	Similarities	S. Sem	Zoo Maps	Parcel
d2-CON	0.673 **	0.746 **	0.577 **	0.529 **	0.428 *	−0.063	0.366 *	0.665 **
HVLT-R-trial3	0.376 *	0.437 *	0.347	0.316	0.366 *	0.053	0.401 *	0.165
HVLT-R-LearT	0.363 *	0.409 *	0.294	0.328	0.468 **	0.02	0.706 **	0.170
HVLT-R-delayed	0.443 *	0.433 *	0.396 *	0.325 ^a^	0.296	0.003	0.507 **	0.247
HVLT-R-recog	0.449 *	0.287	0.366 *	0.355 **	0.224	0.088	0.663 **	0.287
Digits	0.677 **	0.427 *	0.414 *	0.318	0.429 *	0.114	0.238	0.366 *
L&N	-	0.662 **	0.567 **	0.519 **	0.544 **	0.046	0.437 *	0.660 **
Matrix	0.414 *	0.371 *	-	0.444 *	0.472 **	0.096	0.638 **	0.482 **
Similarities	0.429 *	0.224	0.544 **	0.24	-	0.207	0.427 *	0.128
Zoo maps	0.238	0.315	0.437 *	0.315 ^b^	0.638 **	0.102	-	0.408 **

Note: * *p* ≤ 0.05; ** *p* ≤ 0.001; ^a^ tendency to significance *p* = 0.07; ^b^ tendency to significance *p* = 0.08; d2-CON = Test d2 concentration index; HVLT-R = Hopkins Verbal Learning Test–Revised (trial3 = trial 3; LearT = learning; recog = recognition); L&N = Letters and Numbers; N&V = Numbers and Vowels; S. Log = Series of Logic; S. Sem = Series of Semantic; Parcel = Parcel delivery.

## Data Availability

The datasets presented in this article are not readily available because the data are part of an ongoing study. Requests to access the datasets should be directed to srute@ugr.es.
